# Drivers of Public Attitudes towards Small Wind Turbines in the UK

**DOI:** 10.1371/journal.pone.0152033

**Published:** 2016-03-24

**Authors:** Cerian Tatchley, Heather Paton, Emma Robertson, Jeroen Minderman, Nicholas Hanley, Kirsty Park

**Affiliations:** 1 Biological and Environmental Sciences, University of Stirling, Stirling, Scotland; 2 School of Biology, Harold Mitchell Building, St Andrews, Fife, Scotland; 3 Department of Geography and Sustainable Development, University of St. Andrews, Fife, Scotland; University of Porto, PORTUGAL

## Abstract

Small Wind Turbines (SWTs) are a growing micro-generation industry with over 870,000 installed units worldwide. No research has focussed on public attitudes towards SWTs, despite evidence the perception of such attitudes are key to planning outcomes and can be a barrier to installations. Here we present the results of a UK wide mail survey investigating public attitudes towards SWTs. Just over half of our respondents, who were predominantly older, white males, felt that SWTs were acceptable across a range of settings, with those on road signs being most accepted and least accepted in hedgerows and gardens. Concern about climate change positively influenced how respondents felt about SWTs. Respondent comments highlight visual impacts and perceptions of the efficiency of this technology are particularly important to this sector of the UK public. Taking this into careful consideration, alongside avoiding locating SWTs in contentious settings such as hedgerows and gardens where possible, may help to minimise public opposition to proposed installations.

## Introduction

The world is currently experiencing a period of anthropogenically driven climate change with global mean surface temperature increasing since the late 19^th^ century, a warming of 0.85 (0.65–1.06)°C between 1880 and 2012 [1]. The potential ecological, social and economic impacts of these changes are profound and widespread. Rises in sea level and changes to precipitation will cause increased flooding in some areas and long-term drought in others, and will put pressure on our ability to produce enough food for a growing global population [2]. It is predicted that by 2050 up to 37% of species will be committed to extinction [3,4]. A comprehensive review of the economic costs of climate change and the associated impact risks suggests that failure to act to mitigate global climate change may cost 5% of global Gross Domestic Product (GDP) each year, whilst taking immediate action to limit climate change is likely to cost much less at around 1% of global GDP each year [5]. Despite this, while 66% of respondents to a UK governmental public attitudes survey were concerned about climate change, only 5% saw climate change as the top challenge facing Britain [6].

The production of renewable power is one component of worldwide efforts to limit the scale and impacts of global climate change. Wind power is a method of electricity generation identified as one of eight key technologies central to achieving the UK government’s target of delivering 15% of the UK’s energy consumption from renewable energy sources by 2020 (Climate Change Act 2008) [7]. The UK has the sixth largest installed wind power capacity in the world at over 11,000 MW, with more wind farms awaiting construction or in planning [8].

Alongside these large wind farm developments, micro-generation of wind power is a growing industry with over 27,450 small and medium wind turbines in the UK between 2005 and 2014, and a generational capacity of 120 MW [9]. There has been similar growth globally with at least 870,000 Small Wind Turbines (SWTs) installed by the end of 2013 [10]. Micro-renewable technologies, such as SWTs, are scaled down versions of standard renewable energy production technologies designed for use where space is limited. They have been utilised by businesses, communities and individual households to both provide their energy needs and to generate an income from feed-in tariffs (FITs). Small wind turbines are legally defined in the UK as having an electricity generation capacity of up to 50kW (Energy Act 2004), however there is no globally accepted definition, with the upper limit of individual countries’ definitions typically ranging from 15-100kW [10]. Within the UK definition there is wide variation in turbine height and design, as it encompasses both building mounted and free-standing SWTs, horizontal and vertical turbine models, and on-grid and off-grid situations [11].

### Attitudes Towards Wind Power

Negative attitudes towards proposed wind farms from the general public are commonly publicised in the media, giving the impression that there is widespread opposition for this technology with negative visual, noise, economic and wildlife impacts often cited. Despite this portrayal, research in the UK and across Europe consistently finds high levels of support for wind power generation [12]. A survey of over 2000 UK households in 2012 found 68% supported onshore wind power, rising to 76% for offshore wind power [6]. Given this high general support for wind power in principle, negative attitudes towards specific wind farm developments are often assumed to be the result of ‘not in my backyard’ attitudes or NIMBYism. However, it has been argued that this oversimplifies complex and varied explanations given by people for opposition to local wind projects, and does little to increase our understanding of attitudes towards wind power [13]. For example research has uncovered unexpected patterns in attitudes such as those living closest to wind farms being more in favour of them once they are operational [14, 15]. This is thought to be the result of greater experience of wind farms allowing people to better evaluate their impacts, with participants in the research often reporting the negative impacts being less than was anticipated. Thus greater familiarity with turbines may improve public attitudes towards them.

The main public concerns about wind power include landscape or visual impacts, wildlife impacts and noise pollution, particularly where there are few local benefits to offset any costs [15]. However to date most research into public attitudes towards wind power has been conducted in relation to large turbines and wind farm developments [12, 15], or has focussed on attitudes towards green power sources in general (e.g. [16, 17]). The nature and location of SWTs differs markedly from these large wind developments. For example, they can be installed in more urbanised environments such as on buildings, factories and in gardens, where the public may be more likely to live and work in close proximity, and can be owned by individuals and local communities [11]. In contrast, large wind farms require relatively remote, open areas and are typically owned by private companies. This makes it inappropriate to extrapolate findings from studies of public attitudes towards wind farms to public attitudes towards SWTs.

### Implications of Public Attitudes for Small Wind Turbine Installations

At present in the UK the majority of SWT installations require planning permission [11]. Despite this, there is currently a lack of national planning guidance specific to SWTs and there can be significant differences in the requirements and restrictions placed on installations between local councils. For example, a survey across local UK councils of when ecological surveys are requested as part of a SWT planning application found they varied from being requested for almost all applications to never being requested except where the installation was within a designated site [11]. Local public attitudes are known to have a key role in determining the outcome of planning applications [18, 19]. A lack of understanding of, and guidance relating to, public attitudes could result in increased antipathy towards SWTs if they are installed in unpopular locations. Equally, it may cause unnecessary rejections of SWT applications and higher levels of decision appeals, which can lead to higher planning application costs, delays in the planning process, and general uncertainties about application outcomes. These are all disincentives to owning a SWT which has implications for the growth of the micro-generation industry and may influence whether government targets for renewable energy generation are met. It is thus vital to better understand what drives public attitudes to SWTs.

Using a nationwide postal survey, we aimed to identify which factors influence public attitudes towards SWTs in the UK. Specifically, we focused on the following questions:

1. What is the degree of acceptance by the UK public of SWTs?
2. How important is the context of SWT installation (e.g. which habitats / areas they are installed in) in determining how acceptable they are?
3. Does concern over climate change influence attitudes towards SWTs?
4. What factors, including familiarity with turbines and demographic factors, influence attitudes towards SWTs?

## Materials and Methods

### Questionnaire Design

The full postal questionnaire is included in S1 Fig. In summary, it consisted of eight pages and was divided into four sections dealing with the following issues: 1) attitudes towards climate change; 2) attitudes towards wind turbines; 3) attitudes towards SWTs in general and in typical settings; and 4) personal details including demographic information. For each of six typical settings for SWTs (on domestic buildings, in domestic gardens, on road signs, in fields, in hedgerows, and on schools premises), respondents were presented with three example photographs and asked to rate the acceptability of SWTs in that setting to them on a balanced five-point Likert-type scale (from very acceptable to very unacceptable). Several other questions employed a similar five-point scale including asking respondents to state how strongly they agreed or disagreed with statements on climate change and typical wind turbine concerns. Space was provided to allow participants to make comments both on specific questions and on the survey topic overall. To limit any order effects [20] two versions of the questionnaire were created; in these the order in which statements were presented for questions 2 and 11 were varied. Similarly, to limit any acquiescence or primacy effect both negatively and positively worded statements were used [21]. The questionnaire was posted with a two-page letter that included a description of SWTs along with a pre-paid self-addressed envelope and an option to complete the questionnaire online if preferred. The online version of the questionnaire was identical to the printed version, barring some minor formatting changes.

A pilot test of the questionnaire was conducted in and around Stirling, Scotland, UK. Forty participants completed the printed version of the questionnaire in the presence of a researcher, who observed them for any apparent difficulties answering any question, and used follow-up questions to test understanding of the questionnaire. The pilot test confirmed the questionnaire took about ten minutes to complete.

A UK address database based upon the white pages directory and births, marriages and deaths register was purchased from www.customlists.net and the 2000 addresses were selected by generating random numbers and taking the address contained in the corresponding database row number. In order for the respondents views to be representative of the UK public as far as possible, the sample (n = 2000) was proportionally stratified by population size of country, and then further into the 10 regions for England [22, 23, 24], so reflecting the actual distribution of the population [25]. To encourage return we followed up with a reminder postcard two weeks later, and completion of the questionnaire gave entry to a prize draw for £50.

### Data Analysis

As the majority of data collected were ordinal, non-parametric statistical techniques were used for analysis. Friedman’s Test was used to assess differences in the acceptability of SWTs in different settings. Post hoc analysis with Wilcoxon signed-ranks was conducted with a Bonferroni correction applied, resulting in a significance level set at *p*<0.003. The mean scores for each respondent across all six settings were used as the measure of level of SWT acceptance in all further analyses. A score for climate change belief and concern was calculated for each respondent by taking the mean of their agreement with six statements regarding climate change (adjusting for negatively worded questions). Whilst data analysis used all five levels for both scores (unless stated otherwise), for ease of reporting scores are simplified to three levels (agree = strongly agree & agree, neutral = neither agree nor disagree, disagree = disagree & strongly disagree) unless stated otherwise. The influence of potential explanatory variables on acceptance of SWTs was tested using an ordinal regression with main effects only [26]. All variables were entered as factors. The starting model included the socio-economic factors age (four levels), gender (two levels), employment status (six levels), education status (five levels) and type of newspaper read (four variables with two levels each: broadsheet, mid-market, tabloid and other). Familiarity with SWTs (three levels: high, medium, low) and presence of turbines within one kilometre of the home (four levels: both small & large, large only, small only, none) were also included as familiarity with turbines has previously been found to influence attitudes to wind farms [15]. Engagement in outdoor activities (two levels: yes, no) was designed to be a reflection of time spent outdoors and connectedness to the environment. Membership of environmental organisations (two levels: member, non-member), alongside education and type of newspaper read, was expected to influence knowledge of, and access to information about, climate change and renewable energy generation. Finally, because of the distribution of responses for climate change belief and concern, respondent score was simplified to three levels (high, medium and low belief and concern) and included in the starting model as this was expected to affect attitudes towards renewable energy generation. In order to use ordinal regressions, mean agreement scores were rounded to the nearest whole number. From a starting model containing all 13 of the explanatory variables outlined above, a model simplification process sequentially removed the variable with the highest *p* value until only variables with *p* values ≤0.1 remained in the model. We also assessed respondents’ voluntary comments and broadly categorised them into types of concern. All statistical analyses were performed in SPSS version 19 [27]. Averages are expressed as means and confidence intervals at the 95% confidence level.

## Results

### Response Rate

Of 2000 questionnaires posted, 335 were returned as undeliverable. Of the remaining 1665 questionnaires, 194 completed questionnaires were returned, a response rate of 12.0%. A further seven responses were removed from some analyses due to questionnaires being incomplete. Fourteen of the questionnaires were completed online. Regional response rate ranged from 7.7% for London to 17.4% for the North East of England. There were no significant differences in response rates between regions (*χ*^*2*^ (11) = 13.5, *p* = 0.26).

### Demographic Statistics

The gender and age structure of our sample was significantly different from that of the UK population (Gender: *χ*^*2*^(1) = 49.6, *p*<0.001; Age: *χ*^*2*^(5) = 170.2, *p*<0.001). Respondents were predominantly male (74.7%) and 65 years of age or older (51.6%) in contrast to 49% male and 21% 65 years or older in the UK population [28]. Only two respondents were under 35 years. In line with this, over half of respondents were retired (55.3%), with 33.7% in formal employment (full or part time). A total of 30 respondents (16.6%) had no formal qualifications, while 58 (32.0%) had a first degree or higher.

### Familiarity with turbines

All respondents were familiar with large wind turbines but 7.7% (± 3.8) of respondents reported they were not familiar with SWTs. Only one respondent owned a turbine, while 4.7% (± 3.0) of respondents had a large turbine, and 10.9% (± 4.4) had a SWT, within 1km of their home.

### Attitudes towards Turbines

Fewer respondents were opposed to having a SWT (25.3% ± 6.1) than a large turbine (52.1% ± 7.0) in sight of their home while 33.5% (± 6.6) and 18.0% (± 5.4) of respondents were in favour of having a small or large turbine respectively in sight of their home (Wilcoxon signed ranks: *Z* = -3.11, *p*<0.01).

More respondents were willing to consider installing a SWT of their own in order to reduce electricity bills (57.9% ± 1.1) than to reduce CO_2_ emissions (47.2% ± 1.0), while 39.6% (± 0.9) of respondents stated that they would not consider installing a SWT. The cost of installation or feeling that SWTs were a poor investment, not living in a suitable location, concern about a negative visual impact, and doubting the efficiency of this method of power generation were the most commonly giving reasons for this (S1 Table).

**Fig 1:**
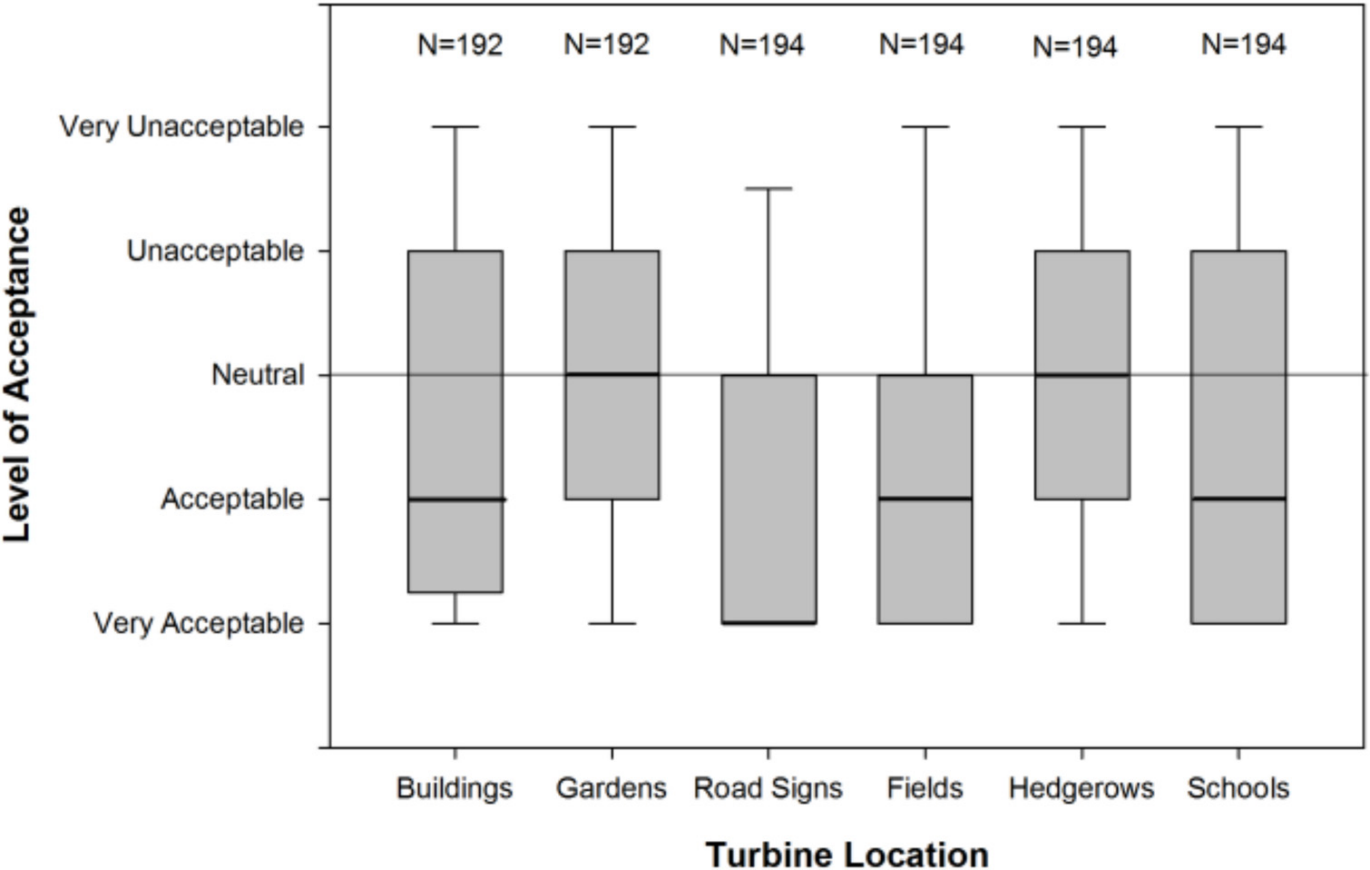
The acceptability of SWTs in different settings. The thick line shows the median while the outer edge of boxes shows 25^th^ & 75^th^ percentile. Confidence intervals represent 10^th^ & 90^th^ percentiles.

The setting of SWTs had a marked effect on the public’s level of acceptance (Friedman Test: χ^*2*^(5) = 126.28, *p*<0.001, Fig 1). Small wind turbines associated with road signs were more acceptable than all other settings presented while those in hedgerows were less acceptable than those on buildings, school premises and in fields, and SWTs in fields were more acceptable than those in gardens (Table 1). Reasons given by respondents for their views on SWT acceptability often focussed on their visual impact (S2 Table). Typically more respondents felt that SWTs had negative than positive visual impacts, with the exception of those on road signs, and those on buildings showed an almost equal split between respondents who felt they had positive versus negative visual impacts. Reasons for the high acceptance of SWTs on road signs were based on the perceived economics, efficiency and practicality of the technology. Noise impacts were not raised as frequently as visual impacts, but when they were the reasons given were largely negative and this is particularly true for the more urban settings of SWTs on buildings, in school premises and in gardens. Some respondents reported needing to know more about noise impacts before they could judge how acceptable SWTs would be in that setting. Overall, concerns over wildlife impacts were relatively few but 31 respondents (16.0% ± 5.2) reported concerns about negative wildlife impacts of SWTs sited in hedgerows. Negative comments about safety were prominent for installations on road signs and school premises but were of little concern elsewhere. The high number of “other” reasons given for SWTs on school premises includes 27 positive and 2 negative comments concerning the potential for education about renewable energy (S2 Table). When respondents’ acceptance of SWTs is averaged across all six settings 50.5% (± 7.0) found them acceptable or very acceptable, while 22.2% (± 5.8) said they were unacceptable or very unacceptable, and the remaining 27.3% (±6.3) were undecided.

**Table 1:** Results of post hoc analysis with Wilcoxon signed-ranks showing pairwise differences in acceptability levels of SWTs in different settings.

SWT Setting	Gardens			Road Signs			Fields			Hedgerows			School Premises		
	N	Z-statistic	P value	N	Z-statistic	P value	N	Z-statistic	P value	N	Z-statistic	P value	N	Z-statistic	P value
**Buildings**	192	-1.723	0.085	*192*	***-5*.*924***	***<0*.*001***	192	-2.968	0.003	192	**-3.055**	**0.002**	192	-1.242	0.214
**Gardens**				*192*	***-6*.*494***	***<0*.*001***	*192*	***-4*.*423***	***<0*.*001***	192	-1.777	0.076	*192*	*-2*.*638*	*0*.*008*
**Road Signs**							194	**-3.428**	**0.001**	194	**-7.975**	**<0.001**	194	**-5.433**	**<0.001**
**Fields**										194	**-6.265**	**<0.001**	194	-1.781	0.75
**Hedgerows**													*194*	***-3*.*747***	***<0*.*001***

Pairwise differences remaining signficant after bonferroni corrections were applied are highlighted in bold. Italic typeface indicates top row setting was more accepted than left column setting.

**Fig 2:**
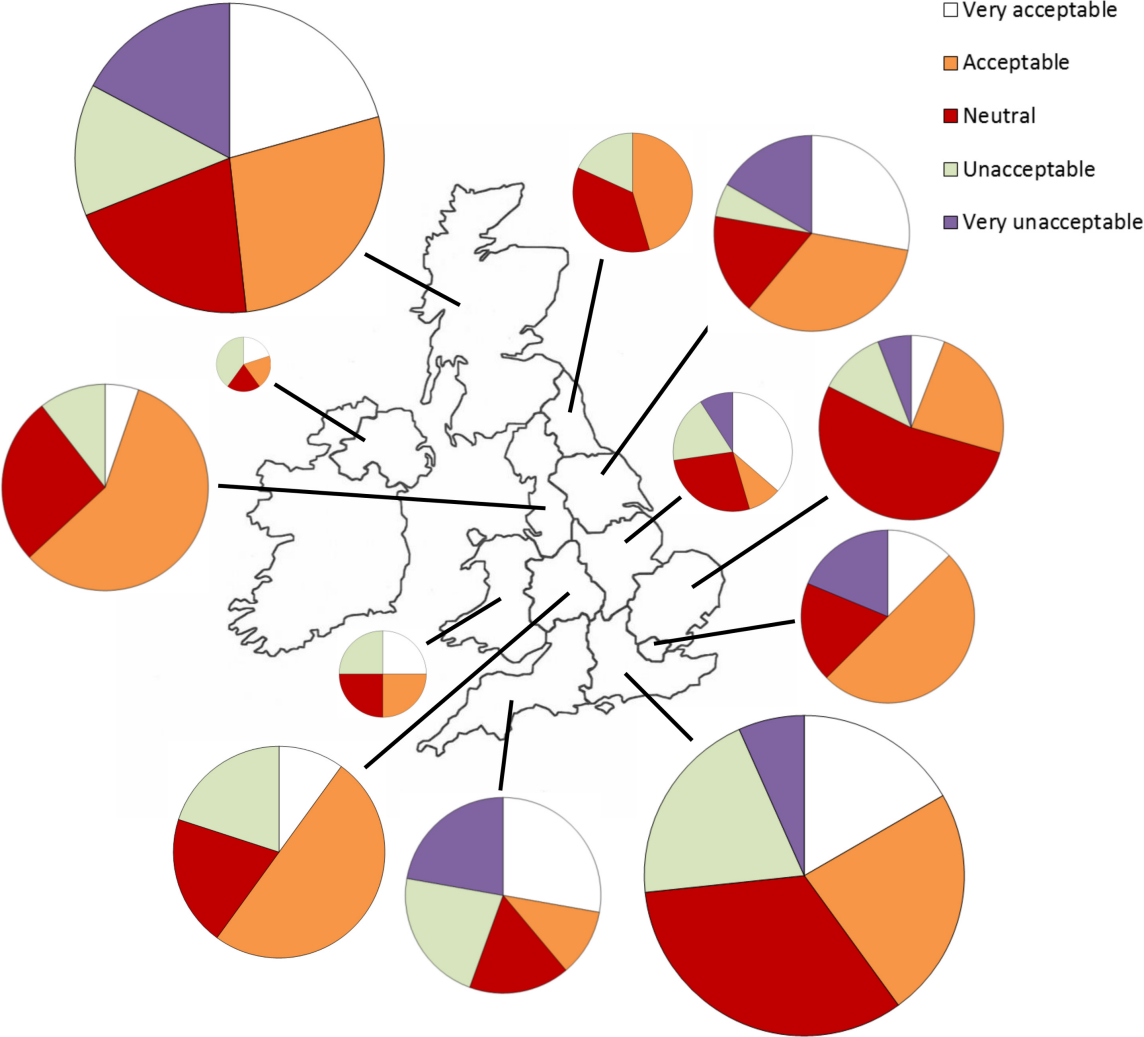
The acceptability of SWTs across the UK. Pie charts show the average acceptance of SWTs across six settings split by region. Numbers are percentages of respondents in each category of acceptance. Size of pie charts reflects the number of respondents from each region.

There were small differences in the acceptability of SWTs between regions, with London and the North West having the highest proportion of respondents who found them acceptable whilst the South West had the highest proportion who found them unacceptable (Fig 2). However these differences were not statistically significant (*χ*^*2*^ (44) = 54.8, *p* = 0.13).

**Fig 3:**
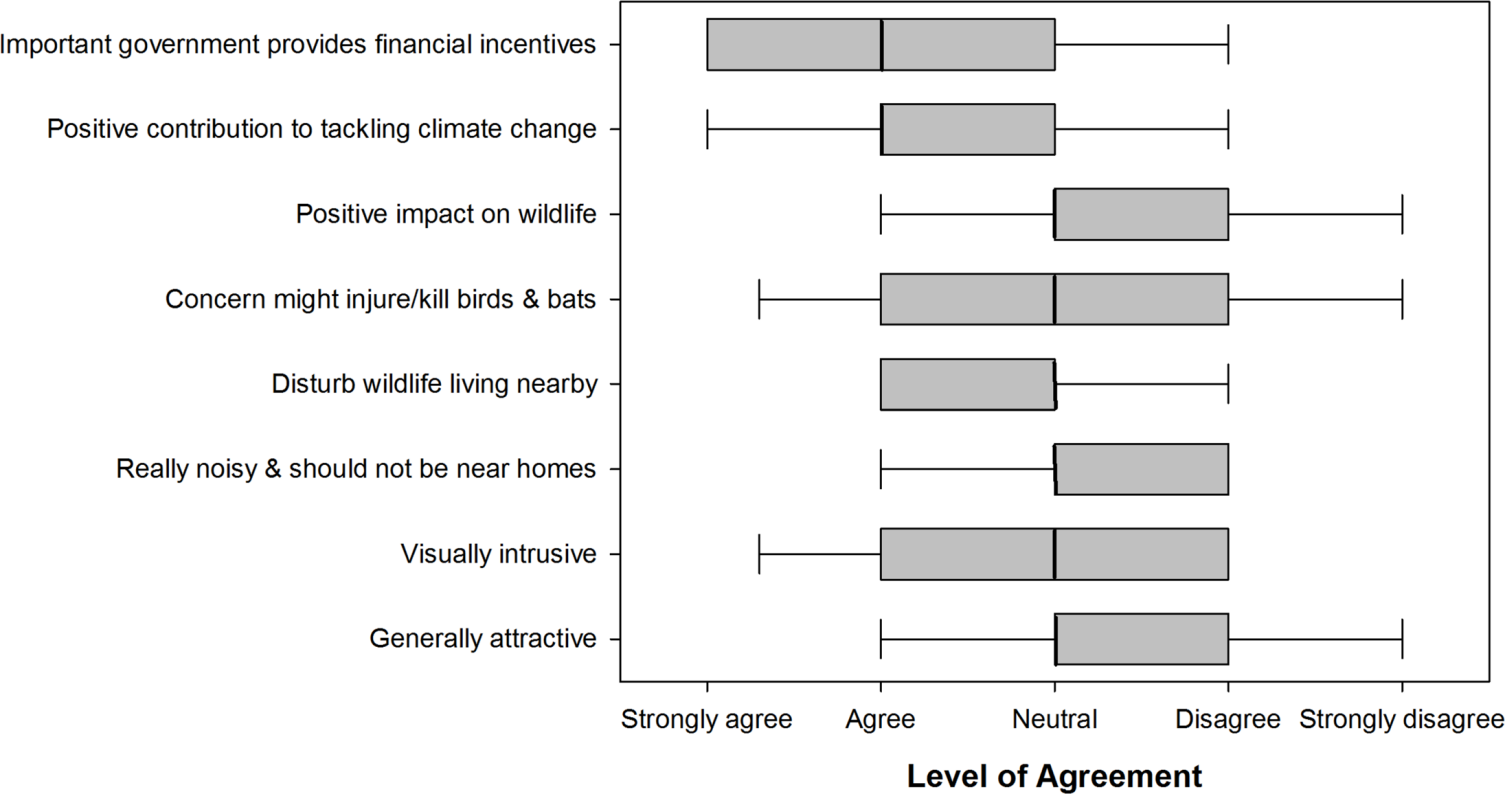
Agreement with statements about typical turbine concerns with regard to SWTs. The thick line shows the median while the outer edge of boxes shows 25^th^ & 75^th^ percentile. Confidence intervals represent 10^th^ & 90^th^ percentiles. N = 192.

Over half of respondents felt that SWTs made a positive contribution to tackling climate change (57.3% ± 7.0), and that the government should provide financial incentives to encourage people to install them (61.3% ± 6.9, Fig 3). Almost equal numbers of respondents felt that SWTs were (30.2% ± 6.5), and were not (34.4% ± 6.7), visually intrusive. There was also little consensus over noise impacts with 22.9% (± 6.0) agreeing, and 30.3% (± 6.6) disagreeing, with the statement that SWTs are really noisy and should not be put up near homes. Over a third (35.4% ± 6.8) of respondents were concerned that SWTs might injure or kill wildlife and 30.7% (± 6.5) felt they would disturb wildlife living nearby. Approximately half of respondents were undecided as to whether SWTs have a positive impact on wildlife (50.0% ± 7.1).

### Attitudes to climate change

The majority of respondents felt they were at least fairly well informed about the causes (80.8% ± 2.8) and consequences (83.8% ± 2.7) of climate change, and the ways we can mitigate against this (70.8% ± 3.3). Very few respondents felt they were not at all well informed on these issues (≤1% for all).

Almost 80.8% (± 5.6) of respondents agreed with the statement ‘we are in a period of global climate change’ and 58.2% (± 6.9) agreed they were worried about climate change, while 28.0% (± 6.3) felt that the seriousness of climate change has been exaggerated. Just over half of respondents (51.6% ± 7.1) disagree with the statement that climate change is an unstoppable process and 81.3% (± 2.8) felt that renewable energy makes a useful contribution to reducing carbon emissions. The mean agreement with these statements was calculated for each respondent as a measure of their level of belief in, and concern about, climate change. This measure was positively correlated with how well informed respondents felt about the causes and consequences of climate change (Spearman’s rank: *r*_*s*_(190) = 0.18, *p* = 0.008). The role of this measure in influencing attitudes towards SWTs was then further explored, alongside other potential drivers of attitudes.

### Factors influencing attitudes towards Small Wind Turbines

**Fig 4:**
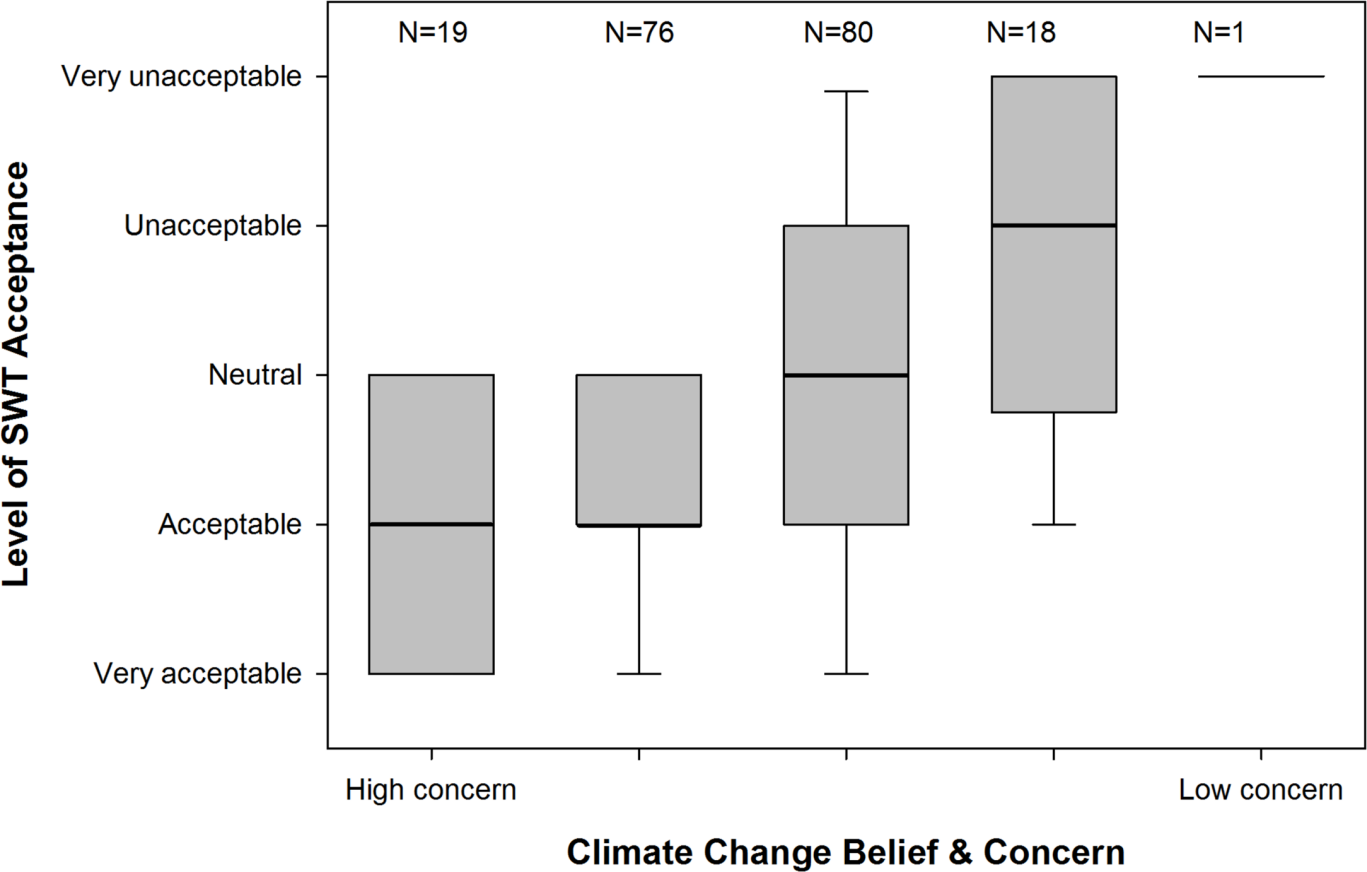
The difference in SWT acceptance between respondents with different levels of climate change belief and concern. The thick line shows the median while the outer edge of boxes shows 25^th^ & 75^th^ percentile. Confidence intervals represent 10^th^ & 90^th^ percentile.

Belief in and concern about climate change, age, and participation in outdoor activities significantly influenced average acceptance of SWTs across all settings (Table 2). Those respondents with high levels of climate change concern were eight times more likely to find SWTs acceptable compared to those with low levels of concern (Fig 4). Respondents who were aged 45–54 years were nearly six times more likely to find SWTs acceptable than those aged 65 years or older. Those who participated in outdoor activities were over nine times less likely to find SWTs acceptable than those who did not take part in such activities. Membership of environmental organisations and readership of midmarket and other newspapers also had an important influence on average acceptance of SWTs. Readers of both midmarket and other (mostly local) newspapers were less likely to find SWTs acceptable than those who did not read these classes of newspaper (two and three times less likely respectively), while members of environmental organisations were almost three times more likely to find SWTs more acceptable than non-members.

**Table 2:** Coefficients and P-values from the final (PLUM) regression model of SWT acceptance across all settings.

Explanatory Variables	Level	Coefficient	SE	Wald	Sig. (P)	Odds Ratio
Climate change belief & concern	High	-2.083	0.760	7.518	**0.006**	0.12
	Neutral	-0.732	0.742	0.972	0.324	0.48
	*Low*					
Age	35–44	-1.766	1.163	2.305	0.129	0.17
	45–54	-1.728	0.686	6.342	**0.012**	0.18
	55–64	-0.420	0.476	0.78	0.377	0.66
	*65+*					
Outdoor Activities	None	-2.224	0.668	11.076	**0.001**	0.11
	*One or more*					
Environmental Organisations	Member	-1.002	0.524	3.656	0.056	0.37
	*Non-member*					
Midmarket Newspaper	Not read	-0.815	0.493	2.733	0.098	0.44
	*Read*					
Other Newspapers	Not read	-0.939	0.482	3.803	0.051	0.39
	*Read*					

Nagelkerke R^2^ = 0.35. A negative coefficient indicates an increase in likelihood of finding SWTs acceptable (acceptance was coded 1 = Very Acceptable to 5 = Very Unacceptable).

## Discussion

In this study we assessed public attitudes towards small wind turbines in the UK, and have identified several potential influential drivers that underlie variation in attitudes. Overall, the acceptance levels of small wind turbines amongst the respondents in our survey was relatively high. However, attitudes towards SWTs differ depending on the type of setting the turbine is installed in, with those in hedgerows and gardens being the least well accepted, and most accepted on road signs. Belief in, and concern about, climate change was related to higher acceptance of SWTs and there is some evidence that membership of environmental organisations is associated with higher levels of acceptance. However, participation in outdoor activities was related to lower acceptance of SWTs and there is some evidence that reading midmarket and local papers is associated with reduced acceptance. Age was also related to acceptance, with those aged 45–54 years being more likely to find SWTs acceptable than older respondents.

### Survey Methodology and Data Limitations

There are a number of strengths and weaknesses to using postal questionnaires as a method of assessing public attitudes. They enable researchers to target a large sample of people efficiently, both in terms of cost and time, when compared to other methods such as telephone and face to face interviewing [21]. However, postal questionnaires can suffer from low response rates, and there is evidence from several countries that response rates to questionnaires may be declining [29, 30]. Low response rates may result in a non-response bias in the sample, where those that have not responded belong to a particular demographic or belief group [21, 29]. This study, which elicited a 12.0% response rate, used follow up contact, the opportunity to respond quickly online and the opportunity to enter a prize draw, methods that are commonly recommended to help maximise response rates [21]. Nevertheless, this low response rate means our sample was biased towards males and older people, and therefore the results presented here cannot be extrapolated to apply to the wider UK population. We were, however, able to survey participants covering a range of educational backgrounds and levels of climate change concern from all regions of the UK enabling the detection of influential variables on SWT attitude and providing interesting and novel insight into attitudes towards SWTs within a section of the UK public.

### Attitudes towards Small Wind Turbines

With the caveat that our sample reflects the views of older generations who are male, the results of this survey suggest a large proportion of this section of the UK public generally finds SWTs acceptable (50.5% ± 7.0) but there is still currently a section of the population that find them to be unacceptable (22.2% ± 5.8), a pattern also seen in attitudes towards large scale onshore wind power in the UK [6].

Despite a general acceptance of SWTs, the majority of respondents would not be in favour of having one in sight of their home (66.5% ± 6.6), although only a quarter would oppose it (25.3% ± 6.1). This could be seen as an example of NIMBYism, and reflects patterns seen in attitudes towards wind farms where proposals for new wind farms may be met with widespread public opposition despite high acceptance of wind power in general. However, looking beyond NIMBYism as an explanation for such patterns, it has been suggested they are examples of a U-shaped development of attitudes [31], whereby attitudes change pre-, during- and post-construction. For example, initially, attitudes are positive to turbines in general but decrease with the announcement of a local development. Possible reasons for this include genuine specific concerns about the proposed development, misunderstandings about the development due to poor communication by the developers, or a retaliation against a perceived lack of fairness and equality in the planning decision process [13]. Once the wind farm is built and the local community becomes familiar with its presence, positive attitudes towards wind farms increase once more to their former levels, or possibly even exceed them. This may be due to the wind farms not having the anticipated negative impacts or they may just become an accepted part of the scenery over time. This suggests familiarity is important to the development of attitudes towards wind power. There is some supporting evidence for this with wind farms; for example, survey respondents living within 1.5km of four proposed wind farm sites around Sheffield, UK were significantly less positive towards wind power development than respondents from matched comparison towns further away from the proposed sites [32], while Scottish surveys of people living in areas with existing wind farms find people living closest to them (within 5km) are most positive about them and most supportive towards the idea of expanding them when compared to those living 10–20 km away [14]. Yet in this study we did not find any relationship between familiarity with SWTs and attitudes towards them. One possible explanation for this difference is that our measure of familiarity focussed largely on whether respondents were familiar with the concept and appearance of SWTs. Very few respondents reported having a local SWT and, given the lack of a centralised database for SWT installations, it is not possible to estimate their proximity to respondent’s homes. Previous research has also demonstrated U-shaped development curves for attitudes towards solitary turbines, but not yet for SWTs [13, 33], so this may be a useful area to focus on in the future.

The landscape setting of an SWT had a substantial effect on the acceptability of the turbine, with those on road signs and in fields being particularly well accepted and, out of the six typical settings covered in this setting, least accepted in hedgerows and gardens. Farmland and gardens are currently the most common locations for SWT installations [11], with farmland turbines often being installed close to hedgerows to minimise disruptions to farm operations, so this may be an area of conflict between public attitudes and current practice. The comments offered by respondents to explain their attitudes illustrates that different settings raised different types of concerns. Comments about the visual impact were prominent across all settings and the majority of respondents felt that this impact was negative, with the exception of road signs where many respondents suggested they visually had no greater impact than the road sign itself, and to some extent SWTs mounted on buildings which were compared by some respondents to TV aerials. The prominence of comments about visual impact corresponds with suggestions that visual and landscape impacts are of most importance to the public with respect to wind farms e.g. [33]. The photos of SWTs on road signs used in the survey were also the smallest examples, suggesting the size of the SWT may influence its perceived visual impact, although it is hard to disentangle effects of size from setting.

There were relatively few comments on the possible wildlife impacts of SWTs, despite 35.4% (± 6.8) of respondents expressing concern that they may injure or kill birds and bats. Small Wind Turbines in hedgerows are the main exception to this and the large number of negative wildlife impact comments raised here (e.g. “Very hazardous for hedgerow animals and birds”), alongside negative visual impact comments, explains the lower acceptance of SWTs in this setting. Negative comments about noise impacts were largely made in relation to SWTs in more urban settings such as on buildings, school premises and in gardens (22.9% ± 6.0 of respondents felt that SWTs should not be put up near homes), although these were less common than comments regarding negative visual impacts. Respondents’ comments also revealed that some concerns are very specific to a setting. For example, SWTs on school premises raised a high number of positive comments about their potential contribution to raising awareness and educating children about renewable power and climate change (e.g. “Good learning about alternative options for energy sources”), a comment not made about the other settings surveyed. Across the six settings explored here, very few respondents rated SWTs as all very unacceptable or all very acceptable. This indicates that attitudes towards, and acceptance of, SWTs is complex and that people may be positive towards wind power and SWTs in general and still have a negative attitude towards SWTs in particular settings, reflective of the apparent discrepancy between high positive attitudes towards wind power and much lower support for local wind developments [19, 34].

We found that there was a considerable degree of uncertainty as to what the actual impacts of SWT may be. These types of comment were highest in relation to wildlife and noise impacts indicating that the respondents to this survey are unclear on what evidence there is for these potential impacts (e.g. “Would they disturb nesting birds?”, “Are they noisy? Cause vibrations?”). This is not surprising given the lack of impartial information available on these impacts of SWTs. For example, there is very little published research attempting to quantify the wildlife impacts of SWTs [35] making it difficult for ecologists and council planning officers to assess the likely impacts of SWTs on wildlife [11]. This suggests the need for further research into the impacts of SWTs, particularly those the public are unclear about, such as noise and wildlife, and that findings should be made easily accessible to the public.

### Attitudes towards climate change

Overall, most respondents (80.8% ± 5.6) did believe in anthropogenically driven climate change and over half of the respondents were worried about it. This is consistent with the results of other recent UK nationwide surveys. The British Social Attitudes survey found 92% of respondents believed climate change is occurring [36] and the UK governmental public attitudes tracker found 66% of respondents were concerned about climate change [6]. Despite this high acceptance of climate change, nearly a third of respondents in our study (28.0% ± 6.3) felt the seriousness of the issue had been exaggerated. Again, this is consistent with other UK surveys with the British Social Attitudes survey reporting 37% of respondents thinking the environmental threats from climate change are exaggerated [37]. Respondents who felt relatively well informed about climate change were more likely to be concerned about it, highlighting the importance of education and access to information, although this could also be the result of those with more concern about climate change choosing to seek out further information.

### Influences on attitudes towards Small Wind Turbines

Our measure of belief in, and concern about, climate change was positively related to acceptance of SWTs across landscape settings. Given that climate change concern was positively associated with how well informed respondents felt about climate change this suggests that greater education and access to information about climate change may increase the acceptance of SWTs in the UK. However, belief in climate change was shown to already be high both in our sample and in other national surveys (e.g. [36]) so there may be limited scope for education to raise belief in climate change to higher levels. Changing attitudes towards environmental issues using education programs is often very difficult and structural solutions, such as changes in government policy that incentivise positive environmental behaviours, are frequently more effective in changing behaviour [38]. Further, opposition to wind farm developments is rarely due to ignorance and as such education is unlikely to change the attitudes of such opponents, whose opposition is often linked to values and beliefs [39, 40].

Very few of the demographic variables we investigated were strongly associated with attitudes towards SWTs. Respondents aged 45–54 years old were six times more likely to be accepting of SWTs than those aged 65 years or older. Given the majority of our respondents were over 65 years our results likely underestimate the wider UK public’s belief in climate change and acceptance of SWTs. Further research surveys targeted at younger age groups, as well as women and people of a wider range of ethnicities, will be needed to investigate this likelihood. Newspapers read were classified into broadsheet, mid-market and tabloid in order of level of seriousness of content with broadsheet papers being those that are perceived as more intellectual in content, tabloids being more sensationalist in content, and the mid-market being inbetween with a mixture of intellectual and sensationalist content. Those who read midmarket newspapers are more likely to have lower acceptance of SWTs than those who do not read this class of newspaper, possibly reflecting a bias in the information on climate change and wind power presented in these papers. Alternately, those who choose to read these papers may already have low acceptance of wind power and choose to read them because they share information that fits their beliefs. Readers of other papers, mostly consisting of local papers, were also more likely to be unaccepting of SWTs. These papers may have greater coverage of local wind power related planning applications and objections. Members of environmental organisations were more likely to be accepting of SWTs but those that participate regularly in outdoor activities were more likely to find them unacceptable, perhaps reflecting concerns that turbines may interfere with these activities through issues around safety and access or through visual and noise impacts affecting enjoyment.

## Conclusions and Policy Implications

The majority of our respondents are accepting of SWTs. However, this general finding does not guarantee acceptance of specific SWT developments for three main reasons. Firstly, acceptance of SWTs was far from universal. Just under a quarter of respondents found SWTs unacceptable with a similar proportion directly opposed to having an SWT in sight of their homes, making it likely there will always be some opposition to proposed developments. Secondly, as has been seen for wind farm developments, a general acceptance may not translate readily into acceptance of a specific development proposal [13]. It is likely that local development proposals will cause concerns about impacts specific to that site even amongst those who are generally accepting of SWTs. Thirdly, our results are biased towards older males and therefore we are unable currently to quantify acceptance of SWT developments within other sectors of the UK public.

There is a need for clearer planning guidance for SWT installations in the UK [11]. The results of this survey provide some useful insights for policy makers, and for developers who wish to minimise the public opposition to a proposed SWT installation. Firstly, the setting of an SWT has been shown to have a significant impact on acceptance so a focus on installing SWTs in more accepted settings such as in fields and avoiding least accepted settings such as hedgerows may help to limit any opposition. Further research looking at acceptance in other settings such as industrial estates may highlight additional well accepted settings. Planning guidance could encourage avoidance of least accepted settings by requiring buffer distances between hedgerows and similar settings as is currently implemented by some, but not all, local councils in the UK, with similar situations elsewhere in Europe. Permitted Development Rights (PDRs) were introduced in Scotland in 2010 (http://www.legislation.gov.uk/ssi/2010/27/pdfs/ssi_20100027_en.pdf) and England in 2011 (http://www.legislation.gov.uk/uksi/2011/2056/made) partly to reduce barriers to the expansion of the micro-generation industry [11]. Permitted Development Rights relax the need for planning permission for those SWTs that meet certain criteria including size and distance to boundary measures, although current guidelines are only likely to affect a small proportion of SWTs being installed [11]. However, there may be scope for PDRs to encourage the installation of SWTs in the most accepted settings, and those least likely to harm wildlife; this could be achieved by modifying the criteria so that planning permission is not required for installations in particular settings, shortening the time and financial costs involved in those installations. Secondly, we have drawn attention to the potential impacts of SWTs that are of most concern to parts of the UK public, namely visual impacts and contrasting perceptions on whether the technology is an efficient and practical method of energy generation. These should be taken into consideration when proposing an SWT installation with steps taken to minimise any negative impacts whilst enhancing potential positive effects; planning guidance should highlight the importance of these factors in particular. Thirdly, the links found between knowledge of, and concern about, climate change and SWT acceptance, alongside the comments from respondents requesting further information on potential SWT impacts, highlights a role for targeted education and easy access to information in increasing acceptance of SWTs across a range of settings.

## Supporting Information

S1 Fig The Questionnaire (DOCX)

S1 Table Summary of reasons volunteered to explain why respondents would not install an SWT at their property showing the number of comments related to each topic and whether they were negative or statements that more information on this potential negative impact is needed before they can decide. Respondents were free to give multiple reasons. A total of 78 respondents (40%) would not install an SWT at their property.
(DOCX)

S2 Table Summary of reasons offered to explain the given acceptability rating of SWTs in different settings showing the % of respondents that made comments related to each subject and whether they were positive, negative or stating they would need to know more about that possible impact before deciding. (DOCX)
