# Supplementary material for: Drivers of Public Attitudes towards Small Wind Turbines in the UK
Source: PLoS One. 2016 Mar 24;11(3):e0152033. doi: 10.1371/journal.pone.0152033 (PMC4806928; doi:10.1371/journal.pone.0152033)
Supplement: S1 Fig — (DOCX) [file pone.0152033.s001.docx]

S1 Fig: The Questionnaire

**Section 1: Your views on climate change**

This section asks about how well informed you feel about, and your views on, climate change.

**1. How well informed do you feel about climate change? Please tick the appropriate boxes for a, b and c below:**

| **How much do you think you know about the following?:** | Very well  informed | Fairly well  informed | Not very well informed | Not at all well informed |
| --- | --- | --- | --- | --- |
| a. The causes of climate change |  |  |  |  |
| b. The consequences of climate change |  |  |  |  |
| c. Ways in which we can fight climate change |  |  |  |  |

1. **Please give your opinions on the following statements concerning climate change by ticking the appropriate boxes:**

|  | Strongly agree | Agree | Neither agree nor disagree | Disagree | Strongly disagree |
| --- | --- | --- | --- | --- | --- |
| a. We are in a period of global climate change |  |  |  |  |  |
| b. Emissions of CO_2_ (Carbon dioxide) has only a small impact on climate change |  |  |  |  |  |
| c. I am worried about climate change |  |  |  |  |  |
| d. Climate change is an unstoppable process; we cannot do anything about it |  |  |  |  |  |
| e. Renewable energy makes a useful contribution to reducing carbon emissions |  |  |  |  |  |
| f. The seriousness of climate change has been exaggerated |  |  |  |  |  |

**Section 2: Your views on wind turbines**

This section asks about your views on wind turbines in general, and then specifically on small wind turbines.

Page 2 of the letter accompanying this questionnaire provides information about small wind turbines and example photographs of a variety of small wind turbines are shown on pages 3 - 5 of this survey

**Large Wind Turbines:**

1. **Are you familiar with what large wind turbines look like?**

Yes € Somewhat € No €

1. **How would you feel towards a large wind turbine installation in sight of your home?**

Very opposed € Opposed € Indifferent € In favour € Very in favour €

**Small Wind Turbines:**

1. **Are you familiar with what small wind turbines look like?**

Yes € Somewhat € No €

1. **How would you feel towards a small wind turbine installation in sight of your home?**

Very opposed € Opposed € Indifferent € In favour € Very in favour €

1. **Do you own a small wind turbine?**

Yes € No €

1. **Are you aware of any large scale (> 30m in height) or small scale (< 30m height) wind turbines within 1km of your home? Tick all that apply**

Large € Small € Neither €

1. **Would you consider installing a small wind turbine on your property for any of the following reasons (tick as many as apply to you):**

Reduce electricity bill €

Reduce CO_2_ emissions €

Other reason ­­­________________________________________________

I would not consider installing a small wind turbine € Please state why:______________________________________________________________________________________________________________________________________________________

**Section 3: Your views on types of small wind turbines**

Below are sets of photographs showing the types of situations in which small wind turbines may be installed.

**10. For each group of photos, please give your opinion on how acceptable you think their use is in this situation (please note you are not being asked to rate each individual photograph).**

1. **Turbines on buildings:**


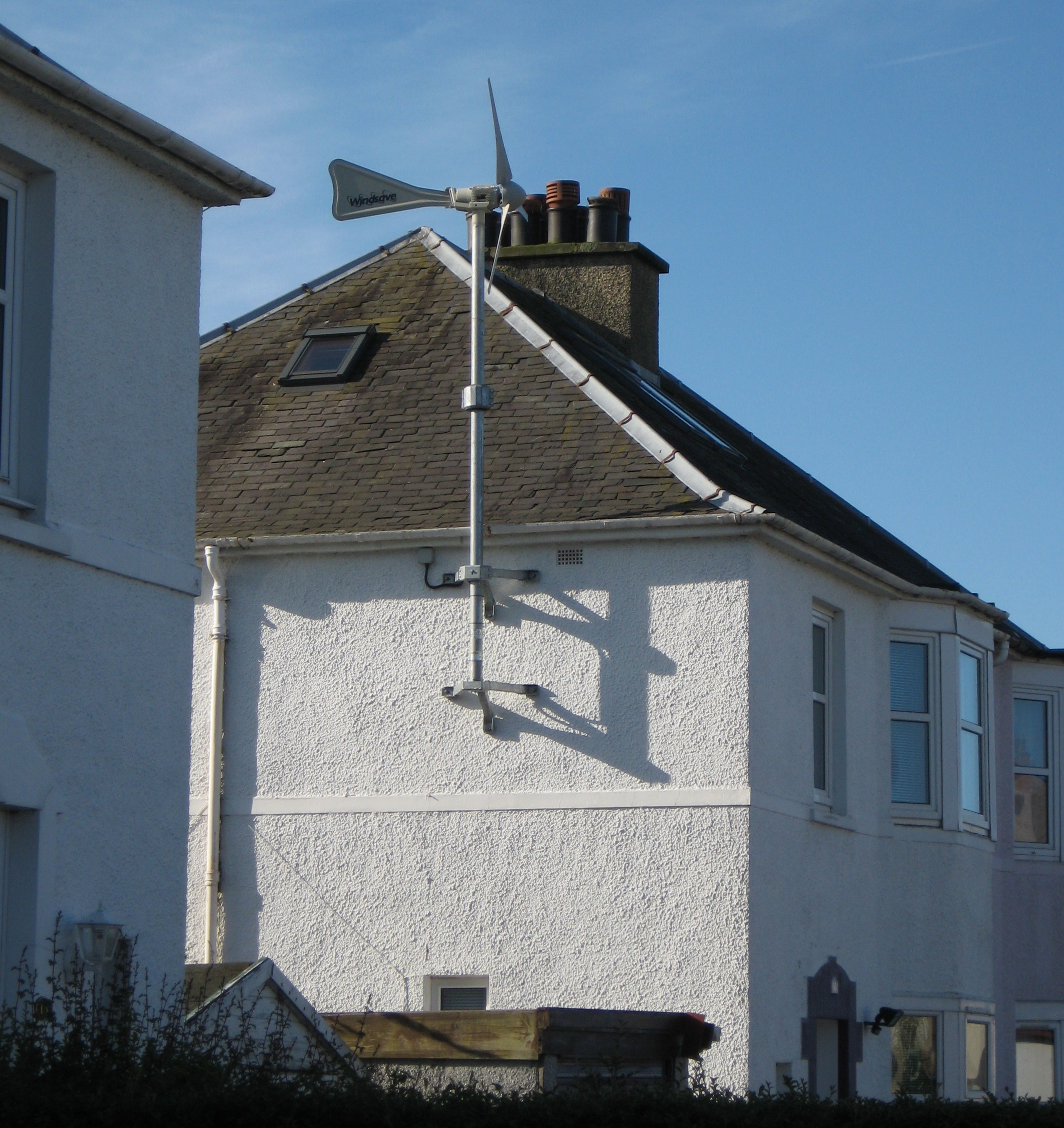


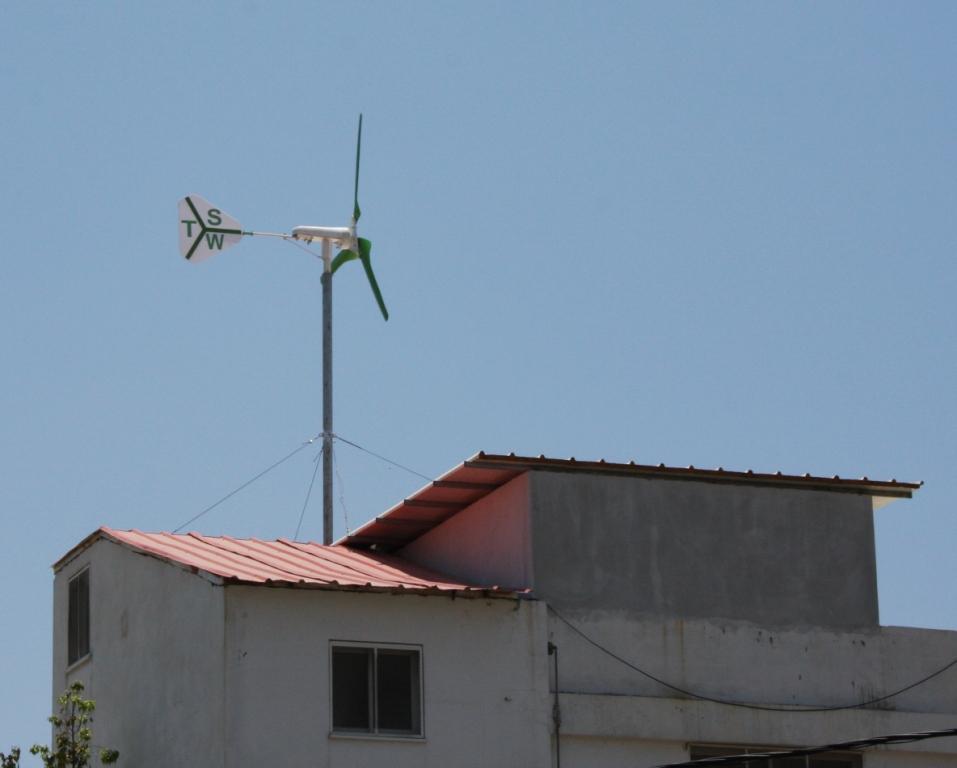


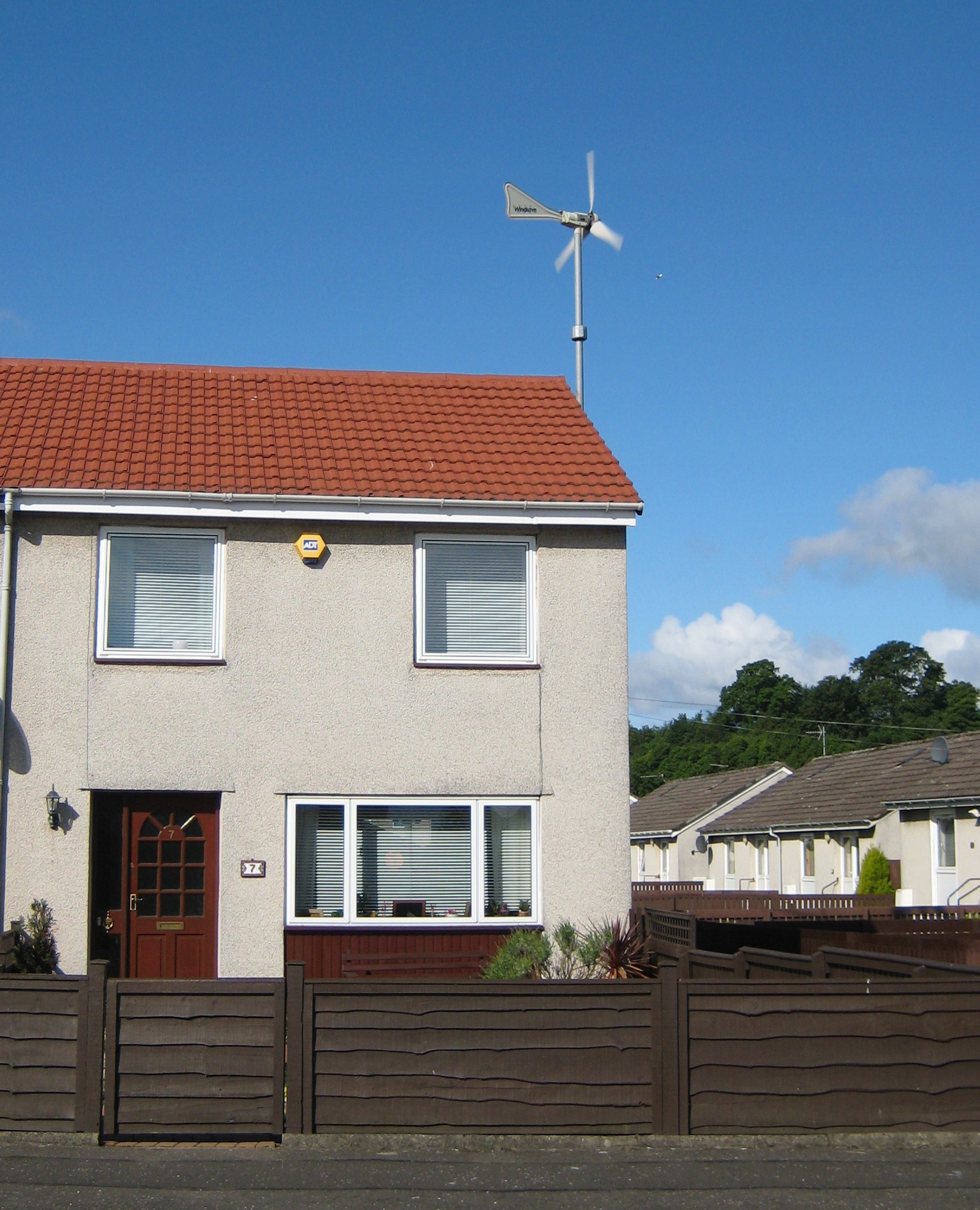


Very Acceptable Very Unacceptable

1 € 2 € 3 € 4 € 5 €

Can you give a reason for your answer?

___________________________________________________________________________________________________________________________________________________________________________________________________________________________

1. **Turbines in gardens:**


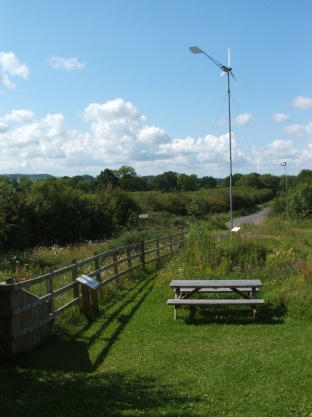

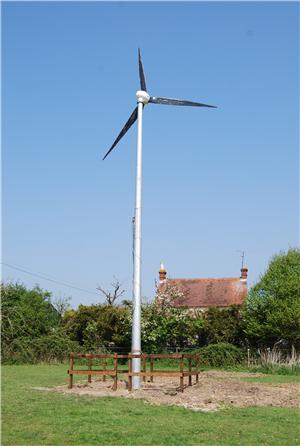

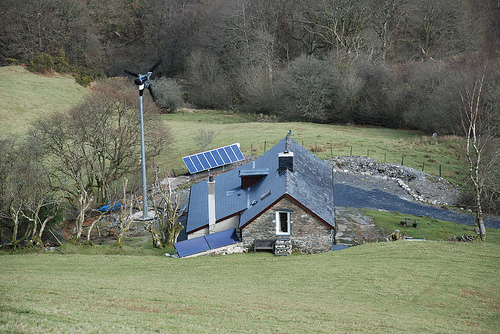


Very Acceptable Very Unacceptable

1 € 2 € 3 € 4 € 5 €

Can you give a reason for your answer? ___________________________________________________________________________________________________________________________________________________________________________________________________________________________


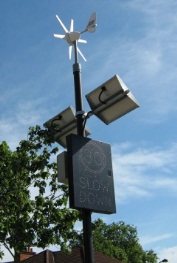

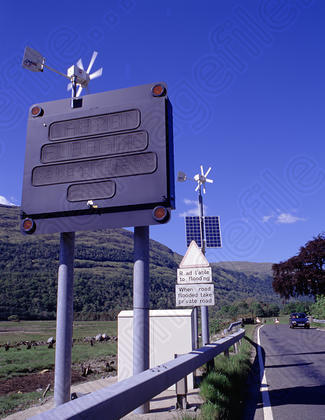

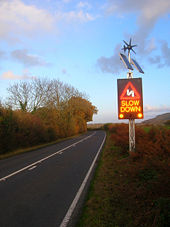
**c) Turbines on road signs:**

Very Acceptable Very Unacceptable

1 € 2 € 3 € 4 € 5 €

Can you give a reason for your answer?

___________________________________________________________________________________________________________________________________________________________________________________________________________________________

1. **Turbines in fields:**


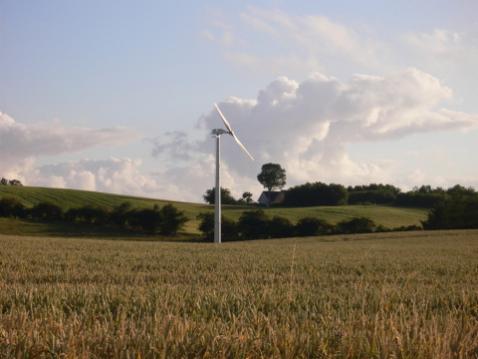

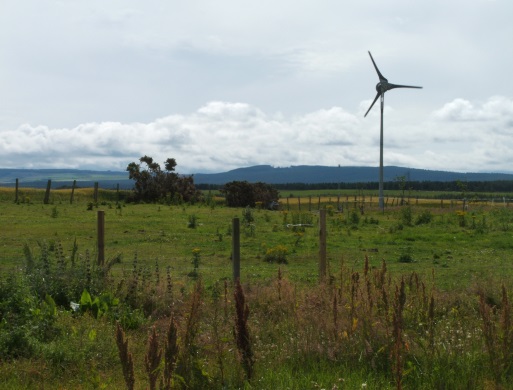

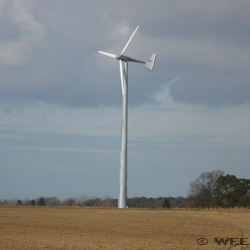


Very Acceptable Very Unacceptable

1 € 2 € 3 € 4 € 5 €

Can you give a reason for your answer?

___________________________________________________________________________________________________________________________________________________________________________________________________________________________

**e) Turbines in hedgerows:**


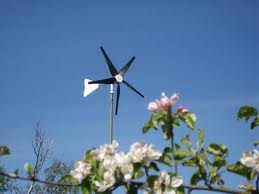


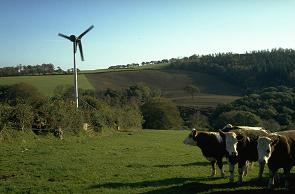


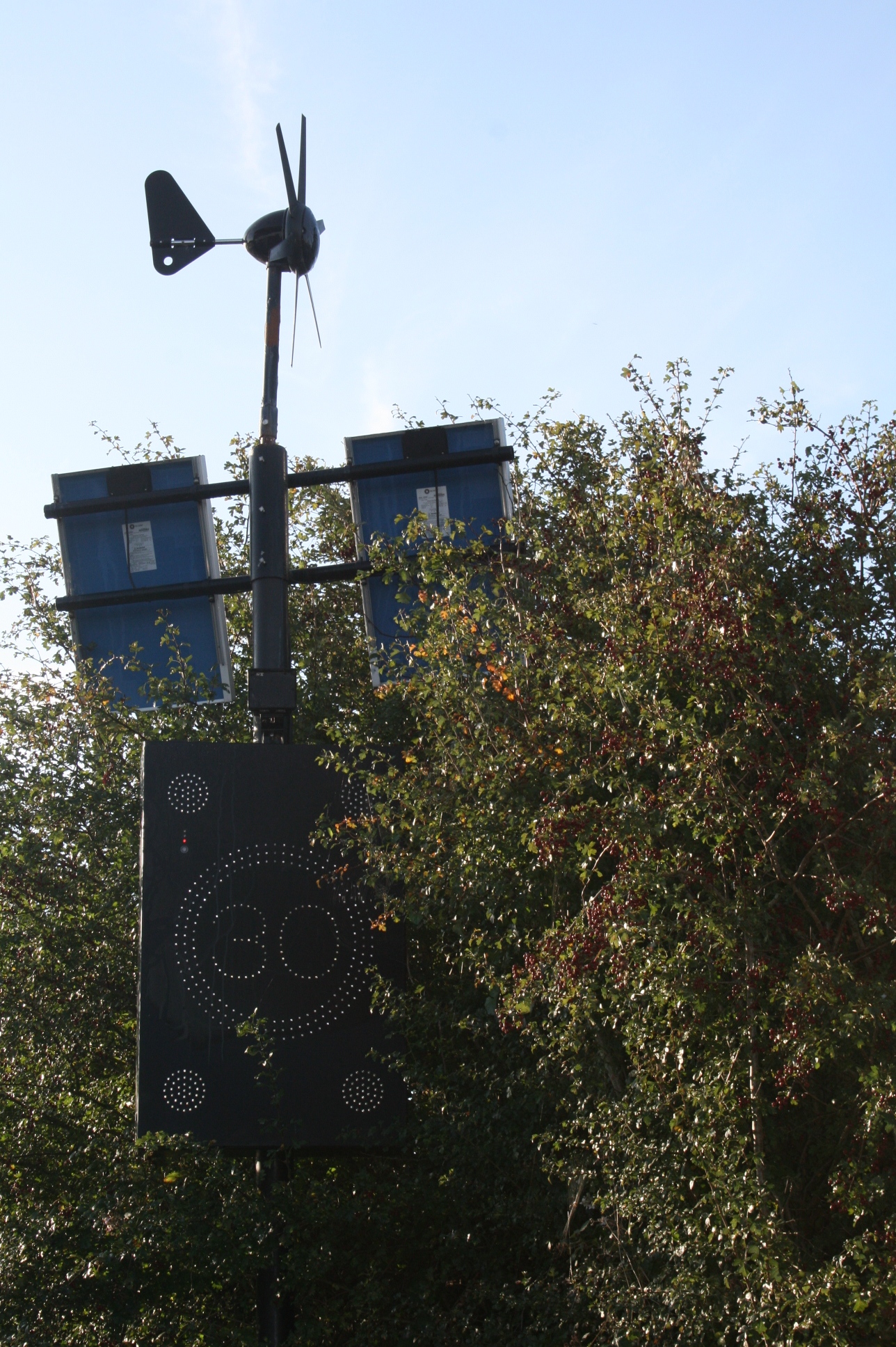


Very Acceptable Very Unacceptable

1 € 2 € 3 € 4 € 5 €

Can you give a reason for your answer?

___________________________________________________________________________________________________________________________________________________________________________________________________________________________

**f) Turbines on school premises:**


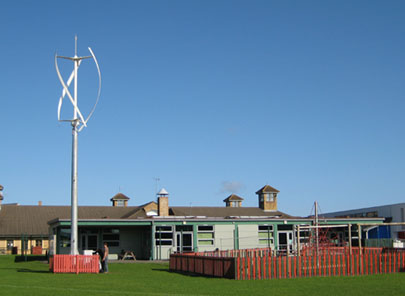


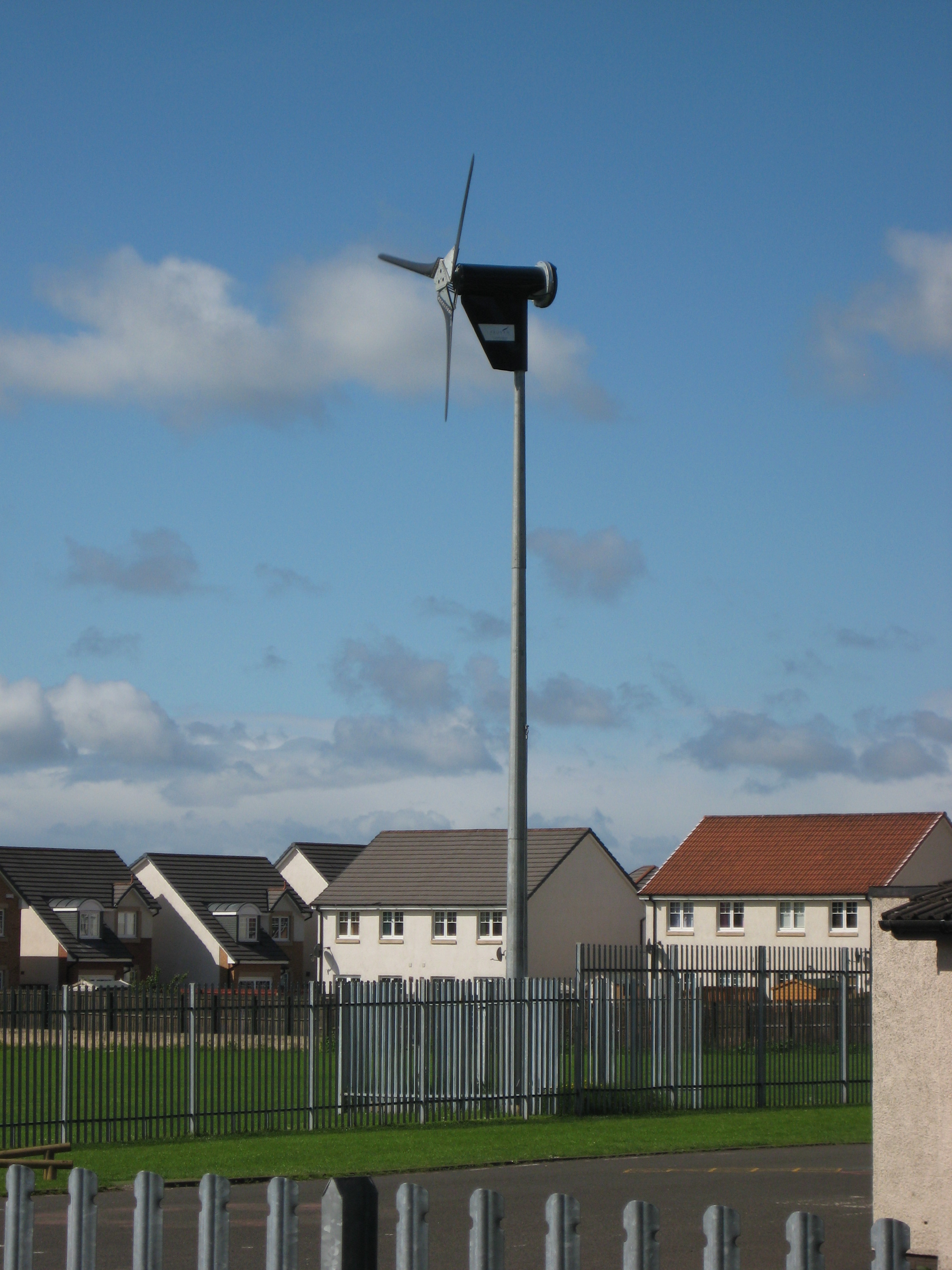


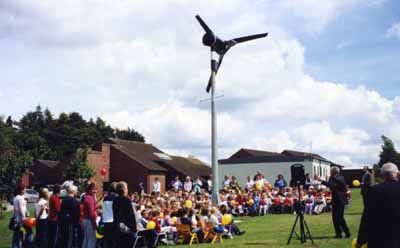


Very Acceptable Very Unacceptable

1 € 2 € 3 € 4 € 5 €

Can you give a reason for your answer? ______________________________________________________________________________________________________________________________________________________________________________________________________________________________

**11. Please give your opinions on the following statements concerning small wind turbines by ticking the appropriate boxes:**

|  | Strongly agree | Agree | Neither agree nor disagree | Disagree | Strongly disagree |
| --- | --- | --- | --- | --- | --- |
| a. I am concerned that small wind turbines might injure or kill birds and bats |  |  |  |  |  |
| b. It is important the government provides financial incentives to encourage people to install small wind turbines |  |  |  |  |  |
| c. Small wind turbines are generally attractive |  |  |  |  |  |
| d. Small wind turbines are really noisy and should not be put up near homes |  |  |  |  |  |
| e. Small wind turbines have a positive impact on wildlife |  |  |  |  |  |
| f. Small wind turbines make a positive contribution to tackling climate change |  |  |  |  |  |
| g. Small wind turbines are visually intrusive |  |  |  |  |  |
| h. Small wind turbines disturb wildlife living nearby |  |  |  |  |  |

**Section 4: About you**

This section seeks general information about you.

1. **What is your postcode?** ___________________________
2. **Are you….?**

Female € Male €

1. **What age are you?**

Under 25 € 25-34 € 35-44 € 45-54 € 55-64 € 65+ €

1. **What is the highest education qualification you have?**

No education qualification € GCSEs / Scottish standard grade or equivalent €

A-levels / Scottish Highers or equivalent € First degree or higher €

Other__________________________________________________________

1. **Which of the following best describes your employment status:**

Full-time paid employment (35 hrs + per week ) €

Part-time paid employment (less than 35 hrs per week) €

Casual employment €

Not currently in paid employment €

Undertaking voluntary work €

Retired €

Other: ___________________________________­­­­__________________________________

1. **Are you a member of any environmental/conservation organisations?**

Yes € No €

If so, which group(s)? ­­­­­­­­­­­­­­­­­­­­­­­­_________________________________________________________

1. **Where do you obtain most information about environmental issues such as…?**

Radio € Television € Friends/Family/Neighbours €

Government bodies € Internet € Environmental Groups € Newspaper € I do not hear about such issues €

Other: ______________________________________

If you regularly read a newspaper (once or more a week) please specify title(s):

­­­­­­­­­­­­­­­­­­­­­­­­­­­­­ ___________________________________________________________________________

1. **Which of the following outdoor activities do you regularly undertake? Tick all that apply**

Walking € Running € Wildlife Watching € Cycling €

Climbing € Shooting € Kayaking/canoeing €

Other_____________________________________________

1. **Do you have any other comments you would like to make?** ___________________________________________________________________________ ___________________________________________________________________________ ___________________________________________________________________________ ___________________________________________________________________________

Thank you for completing the questionnaire. Please return using the self-addressed envelope enclosed.

If you would you like to be entered into the prize draw please fill in your name and email address (if available^1^) below and tick this box €

If you would like to find out the results of this survey please fill in your name and email address (if available^1^) below and tick this box €

^1^If you are unable to provide an email address we will contact you by post.

Name: _________________________________________________

Email: __________________________________________________
