# Supplementary material for: Drivers of Public Attitudes towards Small Wind Turbines in the UK
Source: PLoS One. 2016 Mar 24;11(3):e0152033. doi: 10.1371/journal.pone.0152033 (PMC4806928; doi:10.1371/journal.pone.0152033)
Supplement: S1 Table — Respondents were free to give multiple reasons. A total of 78 respondents (40%) would not install an SWT at their property. (DOCX) [file pone.0152033.s002.docx]

**S1 Table: Summary of reasons volunteered to explain why respondents would not install an SWT at their property showing the number of comments related to each topic and whether they were negative or statements that more information on this potential negative impact is needed before they can decide.**

| **Type of Comment** | **Negative** | **Need Information** |
| --- | --- | --- |
| Noise | 7 | 1 |
| Visual | 22 | 0 |
| Wildlife | 4 | 0 |
| Economics | 24 | 2 |
| Efficiency | 14 | 1 |
| Location/Space | 16 | 0 |
| Safety | 0 | 1 |
| Neighbours | 10 | 0 |

Respondents were free to give multiple reasons. A total of 78 respondents (40%) would not install an SWT at their property.
