# Supplementary material for: Drivers of Public Attitudes towards Small Wind Turbines in the UK
Source: PLoS One. 2016 Mar 24;11(3):e0152033. doi: 10.1371/journal.pone.0152033 (PMC4806928; doi:10.1371/journal.pone.0152033)
Supplement: S2 Table — (DOCX) [file pone.0152033.s003.docx]

**S2 Table: Summary of reasons offered to explain the given acceptability rating of SWTs in different settings showing the % of respondents that made comments related to each subject and whether they were positive, negative or stating they would need to know more about that possible impact before deciding.**

| Turbine Location | Type of Comment | Noise | Visual | Wildlife | Economics, Efficiency & Practicality | Safety | Climate Change & Greenhouse Gases |
| --- | --- | --- | --- | --- | --- | --- | --- |
| On buildings | Positive | 0.5 | 18.8 | 0.0 | 1.6 | 0.0 | 6.3 |
|  | Negative | 6.8 | 18.2 | 0.0 | 1.6 | 0.5 | 0.5 |
|  | Need more information | 4.2 | 1.0 | 0.0 | 0.0 | 0.0 | 0.0 |
| In gardens | Positive | 1.0 | 13.0 | 0.0 | 2.6 | 0.0 | 4.2 |
|  | Negative | 5.2 | 21.4 | 0.5 | 1.0 | 0.5 | 0.0 |
|  | Need more information | 2.6 | 1.6 | 0.5 | 0.0 | 0.0 | 0.0 |
| On road signs | Positive | 1.5 | 16.5 | 0.0 | 16.0 | 1.0 | 3.6 |
|  | Negative | 0.0 | 2.1 | 0.0 | 2.6 | 7.2 | 0.0 |
|  | Need more information | 1.0 | 0.5 | 0.0 | 1.5 | 2.1 | 0.0 |
| In fields | Positive | 0.5 | 9.3 | 0.0 | 2.6 | 0.0 | 2.6 |
|  | Negative | 2.6 | 17.5 | 2.6 | 4.1 | 0.0 | 0.5 |
|  | Need more information | 0.0 | 1.0 | 1.5 | 1.0 | 0.0 | 0.5 |
| In hedgerows | Positive | 0.5 | 7.2 | 0.5 | 2.1 | 0.0 | 2.6 |
|  | Negative | 1.0 | 12.4 | 16.0 | 4.1 | 0.5 | 0.0 |
|  | Need more information | 0.0 | 0.5 | 4.6 | 0.5 | 0.5 | 0.0 |
| On school premises | Positive | 0.0 | 3.6 | 0.0 | 9.3 | 0.5 | 3.1 |
|  | Negative | 7.7 | 7.2 | 0.0 | 2.6 | 8.2 | 0.0 |
|  | Need more information | 2.6 | 0.5 | 0.0 | 1.5 | 3.1 | 0.0 |
